# Stuck between a rock and an unnecessarily hard place—a qualitative interview study of experiences of psychiatric inpatient care among persons with severe dissociative states

**DOI:** 10.1080/17482631.2026.2700066

**Published:** 2026-07-08

**Authors:** Anja Söderberg, Git-Marie Ejneborn Looi, Britt-Marie Lindgren, Josefin Bäckström, Sebastian Gabrielsson

**Affiliations:** a Department of Health, Education and Technology, Luleå University of Technology, Luleå, Sweden; b Department of Nursing, Umeå University, Umeå, Sweden; c Department of Medical Sciences, Clinical Psychiatry, Uppsala University, Uppsala, Sweden; d Department of Health Care Sciences, Marie Cederschiöld University, Stockholm, Sweden

**Keywords:** Psychiatric inpatient care, severe dissociative states, nursing, retraumatization, qualitative content analysis

## Abstract

**Introduction:**

Persons with severe dissociative states constitute a vulnerable group in psychiatric inpatient care and are at risk of being misunderstood and mistreated. The aim was to describe experiences of psychiatric inpatient care among persons living with severe dissociative states.

**Methods:**

Sixteen persons with lived experience of severe dissociative states and previous contact with psychiatric inpatient care participated in semi-structured interviews that were analyzed using qualitative content analysis. This study is reported in accordance with the COREQ guidelines.

**Results:**

Participants' experiences are described in one theme, *Stuck between a rock and an unnecessarily hard place*, and three subthemes. *At the mercy of others in a place intended to provide support, Distrusted and rejected in a place intended to allow existence* and *Lacking control in a place intended to be safe.*

**Discussion:**

The results suggest a gap in understanding of trauma dynamics in psychiatric inpatient care, implying a need for increased trauma awareness. Mental health nurses should lead the promotion of safe, person-centred and trauma-informed psychiatric inpatient care for persons with severe dissociative states, requiring authority, education, support, supervision and resources.

## Introduction

Psychiatric inpatient care (PIC) is an important link in the continuum of care as it can offer a safe place for persons who are struggling on their own and need support (Tully et al., 2023). However, the risks of persons coming to harm (Söderberg et al., 2024; Staniszewska et al., 2019; Tully et al., 2023) and of staff not knowing what to do (cf. Gabrielsson et al., 2016; Söderberg et al., 2022; Wyder et al., 2017) indicate a need for increased knowledge in the field.

Persons in PIC may benefit from the care provided, but they also risk being harmed (Staniszewska et al., 2019; Waldemar et al., 2018) and may even be traumatized or retraumatized through unhelpful or harmful practices (Hennessy et al., 2023; Khatib et al., 2018; Wu et al., 2020) prompting calls within mental health nursing for a paradigm shift towards person-centred and recovery-oriented approaches grounded in theoretical perspectives, such as trauma-informed care (TIC) (Isobel, 2016). The limited knowledge of the experiences of PIC among persons living with SDS indicates a need for further knowledge (cf. Parry et al., 2018; Söderberg et al., 2024).

## Background

Dissociation encompasses a range of symptoms, including depersonalization and derealization, with dissociative identity disorder (DID) often described as representing the most severe manifestation of dissociative states (Snyder, 2021). Dissociative symptoms are reflected across several diagnostic categories, and persons may be misdiagnosed or experience delays in receiving an accurate diagnosis. For this reason, the term severe dissociative states (SDS) is used in this study to refer to experiences commonly associated with DID, rather than focusing on the diagnosis itself. According to DSM-5, DID is characterized by a disruption of identity involving two or more distinct personality states, accompanied by marked discontinuity in the sense of self and agency, as well as recurrent amnesia for everyday events, important personal information and/or traumatic experiences (American Psychiatric Association, 2022).

Different explanatory models to explain SDS exist. Research has established that SDS are related to experiences of severe childhood trauma while also acknowledging that familial, societal and cultural factors may give rise to the trauma and that they may influence the expression of the states (Dorahy et al., 2014; Sar et al., 2017) while other explanatory models, such as the sociocognitive model acknowledges that trauma can be a part of the aetiology but does not assume a direct relationship between dissociation and childhood trauma and that dissociation can depend on influence from media, sociocultural beliefs and suggestive techniques from practitioners (Bachrach & Huntjens, 2025). Sar et al. (2017) describe that multiple self-states that do not become integrated over time allow a child to compartmentalize trauma-related cognitions and overwhelming and conflicting feelings of betrayal, terror, love and shame. Further, dissociative identities and the different bundles of owned memorized experience that characterize them may differ markedly from each other on lower order characteristics such as physiological arousal (e.g., heart and respiration rate, blood pressure), affective tone and neurobiological correlates (e.g., dominant brain activation) and higher-order characteristics, such as the ability to have ownership of lived experience, as well as appraise and narrate them (Sar et al., 2017). Contemporary research suggests that dissociative experiences may be better understood in terms of metacognitive processes and beliefs about memory and the self, rather than as the presence of multiple compartmentalized parts with inter-identity amnesia (e.g Bachrach & Huntjens, 2025; Donath et al., 2025a, 2025b). It has also been argued that there is a need for a transtheoretical framework as current explanatory models are viewed as insufficient (Lynn et al., 2022).

In this study, a phenomenological perspective is adopted, drawing on lived experience and participants' own descriptions of their experiences, rather than relying on a single explanatory model or making claims about underlying mechanisms or psychological structure. From a lived experience perspective, people often describe experiences of SDS using terms such as “having parts,” “being in parts,” “having alters,” or “being split.” The terminology used in this study is understood as reflecting lived experience and meaningful ways of organizing and expressing these experiences.

Increased screening for complex traumatization and dissociative symptoms may be essential for effective treatment (Benincasa et al., 2024). Supportive treatment for persons living with SDS may include psychoeducation, which has been described as a supportive component of care (Pierorazio et al., 2024). Within nursing practice, interventions have been suggested that focus on helping persons reconnect with the present moment, supporting emotional regulation, promoting sleep and physical well-being and addressing safety-related concerns (Snyder, 2021). A range of psychotherapeutic approaches have also been applied in the treatment of SDS. One commonly used approach is phase-oriented treatment (Bachrach & Huntjens, 2025; Griffiths et al., 2025) and in addition, therapeutic approaches originally developed for other psychiatric conditions, such as PTSD and personality disorders, have been adapted for treatment of SDS. These approaches include dialectical behaviour therapy, cognitive behaviour therapy, unified protocol and schema therapy (Bachrach & Huntjens, 2025). The prevalence of DID has been estimated at approximately 1% in the general population, with higher prevalence rates reported in clinical settings (Dorahy et al., 2014). Although research examining the prevalence of dissociative disorders by gender is limited, available studies have not identified clear differences in prevalence between women and men (Foote et al., 2006; Kate et al., 2020). However, SDS are frequently subject to controversy and stigma, putting persons living with SDS at risk of going undiagnosed (Boyer et al., 2022; Snyder, 2021), being misdiagnosed (McAllister, 2000; Snyder, 2021) and being misunderstood due to insufficient knowledge and awareness (Kumar et al., 2022; Parry et al., 2017).

Admissions to PIC guided by treatment guidelines for persons with complex trauma-related symptoms are linked to improvements in adaptive functioning and emotional regulation (Benincasa et al., 2024). Persons experiencing SDS may need PIC to maintain personal safety (Snyder, 2021) in the context of increased risk of self-harm and suicide (Brand et al., 2019; Parry et al., 2017). PIC comprises 24-hour facilities that are staffed institutions in which persons reside following decisions made by professionals and where daily life is governed by institution-specific rules and routines (Topor et al., 2016). The delivery of PIC has changed substantially during the past century, but it continues to be criticized for the dominance of a problem-focused, clinical perspective (Hallberg et al., 2025). Patients' experiences of PIC include feeling safe and supported (Lindgren et al., 2015; Tully et al., 2023; Waldemar et al., 2018), but also loneliness, violation, loss of control (Lindgren et al., 2015; Staniszewska et al., 2019; Waldemar et al., 2018) and a narrow emphasis on acute, symptom-focused stabilization (Waldemar et al., 2019). High-quality PIC has been described as person-centred and recovery-oriented, reflecting theoretically grounded practice models (Gabrielsson et al., 2020; Salberg et al., 2019; Waldemar et al., 2019) and implying a need for contemporary models of nursing. TIC, building on similar theoretical underpinnings, has gained increasing interest and is regarded as a preferred approach for PIC for persons with trauma experiences (Isobel et al., 2021; Isobel, 2016; O’Dwyer et al., 2021; Wilson et al., 2017).

According to first-hand reports, SDS can be experienced as a way of coping with painful experiences (Parry et al., 2017, 2018; Söderberg et al., 2024), managing internal conflicts (Marais et al., 2023; Söderberg et al., 2024) and seeking meaning in unsupportive surroundings (Söderberg et al., 2024). PIC has been associated with feelings of disbelief and exposure (Parry et al., 2017, 2018; Söderberg et al., 2024), whereas outpatient care has been described as fostering safety and trust (Söderberg et al., 2024), highlighting both the potential of supportive care and the risks of mishandled care.

### Rationale

While mental health nursing in PIC is meant to be person-centred and recovery-oriented, actual experiences of PIC vary widely: it may support recovery, hinder it or even lead to harmful outcomes. Persons living with SDS constitute a particularly vulnerable group in PIC, as they are at risk of being misunderstood and exposed to inadequate care. Because research on their experiences remains sparse, more knowledge is needed to better understand their needs and to inform care development.

### Aim

The aim of this study was to describe experiences of psychiatric inpatient care among persons living with severe dissociative states.

## Methods

We used a qualitative design which is appropriate for generating in-depth data and gaining insight into a specific phenomenon (Kim et al., 2017). Guided by the theoretical principles of trauma-informed research and the risks of harm in PIC, we adopted a trauma-informed approach throughout the research process (cf. Isobel, 2021). By working closely with a non-governmental organization (NGO) representative with personal experience of SDS, and by utilizing methodological rigour to centre the participants' voices in the analysis, we aimed to establish a process grounded in respect, safety and shared understanding (cf. Isobel, 2021). This study is reported in accordance with the COREQ guidelines for explicit and comprehensive reporting (Tong et al., 2007).

## Participants

Using purposive sampling (Higginbottom, 2004), we recruited participants with lived experience of SDS and PIC. We included persons with self-reported diagnoses, self-reported experiences of SDS and experiences of any form of PIC. No prior relationships existed between researchers and participants. Recruitment was conducted in collaboration with the above-mentioned NGO via their social media platforms. As recruitment was conducted through social media platforms in collaboration with the NGO, the number of persons reached by the recruitment material is unknown. Sixteen persons aged 22–66 years who met the inclusion criteria participated in the study. The inclusion criteria were persons with self-reported diagnoses or self-reported experiences of SDS and experiences of any form of PIC. There were no specific exclusion criteria. All participants who volunteered took part in the study. The sample size and collected data were considered sufficient to capture variation in experiences and to provide enough depth to address the aim of the study. PIC experiences varied from a few weeks to several years and included voluntary and involuntary, child- and adolescent, forensic, general and acute psychiatry. Fifteen participants identified as women and one as non-binary. Thirteen participants reported being diagnosed with a dissociative disorder. Participants lived in various rural and urban areas of Sweden.

### Data collection

Data were collected through individual semi-structured interviews on a single occasion for each participant (cf. Kvale & Brinkmann, 2014) conducted by the first author, a female PhD student and experienced mental health nurse. The author had no contact with participants prior to the interview and no one else was present during the interviews. While not pilot-tested, the interview guide was developed in collaboration with the NGO representative and guided by the theoretical frameworks of TIC (Isobel & Edwards, 2017; Isobel, 2016) and recovery-oriented care. Participants were given oral information about the aim of the study and were then invited to share their experiences through open questions (e.g., *How would you describe a day in PIC? How would you describe your relationships with staff in PIC?)* and elaborate on their answers. Interviews were audio-recorded and transcribed verbatim. Interviews lasted between 54 and 164 minutes and were performed via Zoom (*n* = 11) and telephone (*n* = 5) between September 2024 and January 2025. Two participants emailed additional information to expand on their answers. To safeguard participants' well-being during the interviews, the information letter encouraged participants to have an updated crisis plan in place in case of distress or retraumatization. At the beginning of each interview, the first author and the participant discussed individual strategies for managing distress and retraumatization should it arise during the interview. Participants were informed that they were not required to answer all questions if they found this distressing. Throughout the interviews, the first author remained attentive to signs of distress and offered breaks or discussion of the interview situation when needed. At the end of each interview, the first author followed up on the participant's well-being, revisited previously discussed coping strategies, and offered the opportunity to make further contact to discuss the interview if needed.

### Data analysis

Inductive qualitative content analysis was chosen to capture manifest and latent content, and the variety of participants' experiences (Graneheim et al., 2017; Lindgren et al., 2020). The whole text of each interview transcript was read repeatedly, then divided into meaning units, which were subsequently condensed and coded. In steps, codes with similar manifest content were grouped into categories and then compared and organized by similarities and differences. This yielded one overarching theme and three subthemes that captured the latent content. The first author conducted all steps of the analysis in continual dialogue with all authors, formulating and refining interpretations through joint reflection. The NGO representative was continually involved and validated the trustworthiness, relevance and plausibility of the results based on lived experience.

### Ethical considerations

We followed the principles of voluntary participation, informed consent and confidentiality. For persons living with SDS, amnesia and difficulties in managing different parts may affect their capacity to consent. Discussing PIC experiences carries the risk of retraumatization. To allow meaningful research participation while mitigating negative consequences, we aimed to create a safe and validating environment (cf. Nester et al., 2023) through a carefully shaped approach developed in close collaboration with the NGO representative. Although sensationalized portrayals of SDS, such as dramatic switches between personalities, have been argued to contribute to stigma surrounding persons living with SDS (Snyder et al., 2024), the terminology used in this study reflects ways in which persons living with severe dissociative states commonly describe and make sense of their experiences. From an epistemic justice and trauma-informed perspective, preserving this language was considered important. This study was approved by the Swedish Ethical Review Authority (ID: 2022-05968-01).

## Results

As presented in Table I and detailed below, our analysis yielded one theme and three subthemes describing the experiences of PIC among persons living with SDS.

**Table I.** Theme and subthemes.

Theme	Subthemes
Stuck between a rock and an unnecessarily hard place	At the mercy of others in a place intended to provide support
	Distrusted and rejected in a place intended to allow existence
	Lacking control in a place intended to be safe

### Stuck between a rock and an unnecessarily hard place

The theme represents an interpretative synthesis of participants' accounts of seeking support in PIC while simultaneously experiencing the environment as in various degrees difficult and harmful. Participants described coming to PIC with hope of finding safety, yet often experiencing encounters with staff as power exercises, invalidating and questioning of their trauma histories and existence. In this context, “existence” referred not merely to physical presence, but to being recognized as a legitimate subject whose experiences, trauma history and parts were acknowledged as meaningful. The theme builds on the English-language idiom “stuck between a rock and a hard place,” referring to a situation in which all available options are experienced as difficult or undesirable. In this analysis, the “rock” represents participants' past, ongoing and anticipated trauma-related challenges outside PIC. The “unnecessarily hard place” represents participants' experiences of PIC as an environment that, although holding the potential to provide safety and support, was experienced as harmful when failing to do so. The term “unnecessarily” reflects participants' accounts of supporting and containing care as a possibility although not consistently a reality. Participants described feeling compelled to choose between managing overwhelming challenges alone outside PIC or risking further harm within it. The subthemes *At the mercy of others in a place intended to provide support, Distrusted and rejected in a place intended to allow one's existence,* and *Lacking control in a place intended to be safe* represent different aspects of the theme.

#### At the mercy of others in a place intended to provide support

This subtheme captures the essence of one prominent aspect of being stuck between a rock and an unnecessarily hard place, emphasizing that while PIC holds support as both a possibility and reality, it was experienced as provided or withheld at the mercy of staff and not something to be counted on. The sense of being in someone else's power evoked earlier experiences of vulnerability and loss of control. Although some staff attempted to provide support and restore a sense of agency, these efforts did not fundamentally alter participants' overall experience of being exposed to others' power, which contributed to feelings of unsafety and risk of further harm. The empirical basis for this part of the interpretative synthesis is clarified below, as we account for how related experiences were articulated by participants.

Participants described how staff exercised power which complicated relationships by creating distance, and they emphasized that those meant to provide care also held power and control over them. This included experiences in which staff were perceived as enemies when they carried out coercive measures and a sense of “us and them.” Although participants described that some staff fostered positive change by communicating trust and focusing on their strengths, they still felt excluded from genuine participation in their care, for example when staff was experienced to “take over” or as ignorant of participants' needs and wishes. Participants described difficulties speaking up, hindering true participation. One participant described being invited to participate but still found it difficult to speak up against educated staffs' suggestions. Participants felt that some staff were unwilling to listen, sometimes acting contrary to their needs and labelling them as “difficult” or “borderline.” Participants described that this had led to dismissal and involuntary discharge. Participants experienced power struggles and for some, reactions on this were described to lead to coercion. For example, one participant described being placed under special observation with exclusively male staff despite having requested female staff. The participant reported that this arrangement preceded an episode of self-harm, which was followed by physical restraint. Participants felt that labels such as “difficult” and “borderline” followed them and for some, this was experienced to routinize coercion and police escorts, and reinforce perceptions of them as dangerous as well as undermining their safety, as one participant described:

*I didn’t really get a chance from the beginning but it started before they even knew a lot about me when I put my nails into my wrist and that was enough for them to rush over and hold me down and then put me in restraining belts. That was only hours from my first contact and then medication that made me be gone for several days. It went so wrong, before they even knew what they treated at all. And then I have experienced that it followed me. That I had to be picked up by the police and use hand cuffs. Since I have had anorexia, I wasn’t strong, I didn’t weigh much, it wouldn’t have been so hard to do it in another way.* Participant 2

Participants described PIC as dehumanizing, and they felt betrayed by the very staff who were expected to provide support. Participants also expressed ambivalence: they were happy to be alive, which they might not have been without PIC, yet one participant expressed a wish that the interventions had been less traumatizing. One participant described experiences of dehumanizing exercise of power:

*If they place me in something that feels like a war, (…) psychiatry, or the so called care, was so much more traumatic than all the trauma I went there for. They have topped what I had problems with from the beginning.(…) It’s so weird, like inside-out weird, I’m actually looking for help and they make sure that they ruin my whole life.* Participant 6

Participants described situations of coercion, which reminded them of being subject to others' violence when staff took over. They described that this caused feelings of fear, exposure, powerlessness and hopelessness, as well as wanting to escape, self-harm and not daring to stand up for themselves. Participants also reported that staff ordered them to stop dissociating to get out of coercion but did not offer any support, which led to feelings of despair. However, being subject to coercion was for one participant experienced as “getting a break” and for another participant responding to a longing for someone else to take control, or as the violent treatment reflecting one’s worth. One participant described being reminded of being subject to others' violence:

*Everything indicates violence. Like all the systems are built on force and violence and like, well we have experiences of trafficking and isolation and being drugged, being tied up, I mean everything that we know that inpatient care and involuntary care deal with are exactly things that we’ve escaped from.* Participant 14

Participants experienced that coercion occurred when staff were unable to handle their strong emotions such as panic and anxiety, self-harm or the present part and participants suggested that they could have acted differently to avoid coercion, as described by two participants:

*Because they approach a scared girl who can’t communicate because she is dissociating heavily with violence, holding down, taking down, dragging and screaming and four, five persons rush over to hold you down.* Participant 2

*They have to be able to adjust themselves to who’s in front of them. It might be someone who’s five years old.* Participant 16

In situations of coercion, participants found staff's behaviour to be dehumanizing, and they wished for a more caring approach. One participant described being placed in restraint belts while staff were scrolling on their phones, rather than attending to the participant or attempting to provide comfort. In contrast, another participant described needing minimal interaction while in restraint, as the situation itself was experienced as so traumatizing that dissociation was necessary. Participants also perceived that staff exercised unnecessary power, such as dragging them away when they were sitting on the floor. Participants further described that they were sometimes left alone, but both practices could be experienced as indicating power and indifference, as one participant described:

*Both when they have left me alone and when they have intruded with all of their interventions it’s been in a way that feels aggressive and mean.* Participant 14

Participants also described that they were met with anger and that they experienced that staff used threats and coercion to control the ward. For example, one participant described experiences of staff invoking the possibility of compulsory admission as a means of behavioural control when perceived as difficult or challenging.

#### Distrusted and rejected in a place intended to allow existence

This subtheme captures another aspect of being stuck between a rock and an unnecessarily hard place, emphasizing that while some staff acknowledged and validated their existence, participants also described encounters characterized by doubt, dismissal or minimization. These experiences were perceived as threatening their existence and belonging within PIC. Validation and invalidation coexisted, yet recurring experiences of disbelief undermined participants' perception of PIC as a place where they could safely exist as they were. The empirical basis for this part of the interpretative synthesis is clarified below, as we account for how related experiences were articulated by participants.

Participants described that they were told that their diagnosis did not exist and felt accused of seeking attention. This included experiences of staff perceiving their dissociation as theatrical or performative. They experienced that their trauma experiences were minimized, which made them feel dehumanized and dismissed and often led to increased self-doubt. Participants felt let down by staff who they expected to be understanding. One participant described that their traumatic history was regarded as implausible by staff. Another participant reported being told that their traumatic memories were insufficient to justify trauma-related symptoms. This participant further described feeling compelled to disclose increasingly detailed aspects of trauma to be believed and worthy of care, yet experienced repeated rejection, including staff closing the door and leaving. The participant described being left with overwhelming emotions and suicidal thoughts. Another participant described being dismissed:

*It can also be like “no you’re exaggerating and you don’t hear any voices and it’s just your thoughts” and again it’s like oh well, am I lying then? Because I’m already unsure of my own experiences as it is and it’s hard when staff say that I’m making it up.* Participant 9

According to participants, staff seemed to prefer some parts of them and made clear that they only wanted to talk to the grown-up, “real” part. In their experience, staff assessed the behaviour of some parts as better, such as talkative, reflective and positive parts. Moreover, participants described that staff misunderstood the switching between parts, and they felt blamed for being defiant and manipulative. Participants experienced that their younger, more emotional parts were told to act like an adult and they described that they received forced medication and electroconvulsive therapy when certain parts showed themselves as one participant described:

*I experienced a lot that they wanted to take parts away from me by medicating away my sad parts. And when I was feeling better they kind of thought that I was nice and they wanted to see more of that one. But the other parts were also me [laughing]. (…) I wasn’t allowed to exist.* Participant 2

Participants felt that they were wrong, unworthy and not allowed, which in turn could lead to despair, hopelessness and escalated self-harm and suicide plans as one participant described:

*He [staff] was angry and provoked and said that he only spoke to (…), there is only her, and that created a huge frustration for that other part when someone said that you don’t exist. You don’t exist, hmm… we don’t talk to you, we don’t listen to you, so it got very impulsive and an act of self-harm where I cut my throat pretty badly.* Participant 16

Participants also described that younger parts were too scared to come forward since the ward environment was not designed to meet their needs, and they feared being misunderstood and ridiculed. To protect themselves, participants described that they shut down parts linked to creativity and vitality, which led to emptiness and depression. However, participants also described feeling validated and allowed to exist when staff believed in them, adapted to different parts and offered non-corrective support with space for everything to exist. For example, one participant recalled how a particular staff member was able to remain present in the face of intense emotions, such as despair, without attempting to suppress, redirect or dismiss them, but instead simply stayed and offered steady presence. This created a sense of safety that was described as challenging but at the same time the only path forward. At the same time, participants also described that certain parts coming forwards could be a result of being in difficult situations where one part was forced to take over.

#### Lacking control in a place intended to be safe

This final subtheme captures a third prominent aspect of being stuck between a rock and an unnecessarily hard place by emphasizing participants' accounts of experiencing limited control within an environment they had hoped would provide safety. Although participants described occasional moments of feeling safe, these were often overshadowed by experiences of unpredictability, perceived lack of knowledge among staff, and an environment described as emotionally triggering. These conditions contributed to a persistent sense of insecurity and a heightened need for control and predictability. Participants further described feeling abandoned when no consistent or reliable sense of safety was available, despite individual instances of supportive care. The empirical basis for this part of the interpretative synthesis is clarified below, as we account for how related experiences were articulated by participants.

Participants described that they longed for control, predictability, educated staff and a kind, accepting atmosphere without coercion. Important aspects of predictability were identified by participants as time, understanding and absence of demands. Furthermore, participants described that they wished to be provided with support according to their needs and easily accessible care. By contrast, participants felt that they were met with routines and few therapeutic activities: the days were described as consisting of waiting and little activity. Participants described this as being “stored” rather than cared for. Participants further described experiences of sexual abuse by staff, along with staff not protecting them from violence from other patients. For example, some participants reported having been physically or verbally attacked by other patients and experiencing that staff were unable to stop the incidents. Such situations reinforced their perception of PIC as unsafe. Participants also described experiences in which staff initiated sexual relationships with them while they were in a vulnerable position and felt unable to refuse. One participant further described that the staff member who initiated such a relationship had previously been the only person who had engaged with her consistently and shown interest in her. Moreover, participants reported that staff made uninformed guesses as to how their trauma background affected them, and they described that they received various diagnoses and felt blamed when treatments did not work. For example, participants described that their dissociative symptoms were interpreted as psychosis or borderline, resulting in the prescription of high doses of antipsychotic medication and they reported experiencing severe side effects. In addition, participants had received dialectical behaviour therapy, which was described as partially helpful but insufficient in addressing their dissociative difficulties. Finally, PIC services seemed inaccessible to participants when they had to argue to be admitted. One participant described experiencing profound despair after repeatedly being refused admission at times when she felt close to dying. This participant further explained that these refusals contributed to a reluctance to seek help within PIC in the future. Although participants experienced some aspects that contributed to predictability and safety, such as some supportive staff and not being able to self-harm, predictable safety seemed out of reach due to the ward environment, past experiences and the complexity of safety. One participant explained that due to prior traumatic experiences, safety required an unusually high level of gentleness and reliability from staff in order to outweigh earlier harm. While some staff succeeded in providing this, the inconsistency across the care environment was described as painful, leading to this participant's conclusion that admission to PIC was not ultimately beneficial. The ward environment and daily routines reminded participants of previous trauma and one participant had been told that they were not meant to feel comfortable there. Participants described inconsistent care shaped by staff's individual approaches and they experienced that certain approaches around self-harm, stress and lack of planning led to perceived punitive or resource-driven discharges. For example, one participant described being required to leave PIC following a panic attack and attempts at self-harm. When this participant was unable to leave the ward due to ongoing panic, the police were called to assist with discharge. After being transported home by the police, the participant reported a subsequent overdose. Participants further described experiencing staff as stressed and eager for them to be discharged before they felt ready. This was described by participants to be linked to both perceptions that admissions should be brief and to limited bed availability. Participants described using compliance as a coping strategy, which they experienced could be misinterpreted as clinical improvement and consequently lead to premature discharge. One participant described how everyday practices reminded of previous trauma:

*It’s such a weird thing when they go into your room at night, like I don’t get it [laughing], like I don’t get how traumatised persons can cope with that but when you’ve been exposed to a lot of violence, a lot at nighttime, it’s like what do they expect to happen when someone sneaks in in the middle of the night and checks you out.* Participant 14

Participants explained that they found their own ways to find control and deal with the ward environment, including engaging in activities, being compliant, adjusting their whereabouts in the ward, having little contact with staff and getting to know the ward rules. One participant described dissociating to handle boredom and traumatic situations. Furthermore, to cope, participants found it necessary to get support from people other than staff, such as other patients, family, friends or outpatient psychiatry. PIC was experienced as unhelpful by participants who felt abandoned or left with limited outpatient support. In relation to this, participants described blaming themselves or feeling punished, and they perceived a lack of responsibility from PIC, involving experiences of PIC not acknowledging that mistakes had been made and one participant further described that formal complaints were dismissed on the grounds of staffs' denial, which fuelled their sense of unsafety. This was experienced to strain participants' relationships with outpatient care, where they could feel forced to lie to avoid readmission, as one participant described:

*And then I have to lie to her. That’s how it is unfortunately because I don’t want to, I get really scared. And it’s sad in a way because in one way I can understand that I need the protection, not for long but still there is a need of safety and that’s because I’m like, for others psychiatry is safety but for me it’s everything but safety (laughing). If I’m unsafe I won’t go to another place where I’m unsafe (laughing).* Participant 12

On the other hand, participants experienced glimpses of good care, with support, structure and coping strategies. Participants felt that they could temporarily let go of responsibility and one participant described PIC as life-saving. This involved accounts of experiencing relief through physical containment and the consistent provision of basic needs, such as sleep, rest and nourishment. Participants felt strengthened by meaningful relationships, both with staff who were knowledgeable about dissociation and staff who, despite limited knowledge, met them with openness and responsiveness. Participants described that when staff genuinely engaged, it fostered trust, hope and a sense of humanity. One participant described these staff members as “gems,” emphasizing that their interactions restored a sense of being recognized as a human being. Nevertheless, participants explained that this was not enough to outweigh the unsafe aspects. One participant described that a particular staff member had been supportive but however, this did not alter the overall experience, which was perceived as unsafe. The participant reflected that the situation might have been different had the same approach been consistently demonstrated by the wider staff group. As described by participants, this could also involve contradictions and unpredictability:

*It was very, very important, at the same time as it gave me hope that I would actually be believed. But at the same time it was very, it got very hard because that didn’t happen. And she was, yes she came to harm. She couldn’t keep openly approaching me the way that she did but she had to do it behind the others’ backs and she tried, I think she fought for me actually.* Participant 10

*Some are more understanding and try to meet the present part but it’s also very confusing because you never know who you meet.* Participant 16

## Discussion

The results describe experiences of PIC among persons living with SDS under one theme, *Stuck between a rock and an unnecessarily hard place*, and three subthemes: *At the mercy of others in a place intended to provide support, Distrusted and rejected in a place intended to allow existence* and *Lacking control in a place intended to be safe*. The results illustrate the dual nature of PIC: a potential source of support, safety and healing that risks evoking previous trauma and confronts persons with the dilemma of either facing life on their own or risking further harm in PIC. These findings may be perceived as unfairly critical of PIC staff, while taking a critical approach to lived experience might be considered diminishing. However, considering different rationales for possibly traumatizing actions and approaches does not invalidate patients experiences, nor does the possibility of a benevolent rationale make the traumatizing potential of these actions less relevant to consider for mental health nursing practice. Simply put—an action or approach might be considered good and necessary from one point of view while bad and unnecessary from another. Only by bringing forth and acknowledging the legitimacy of multiple perspectives can we develop a truly evidence-based practice. We believe that an increased understanding of when and how certain actions and approaches might cause patients distress is for the benefit of patients and staff alike. That said, neither participants accounts, the interpretations we made in the analysis, or the conclusions drawn here suggest that participants negative experiences of PIC is best understood as the results of staff having malicious intent. Rather, we consider it more meaningful to explain these findings in terms of trauma-related vulnerability and potential retraumatization, and suggest that they can best be understood as results of the organizational and structural conditions that shape clinical practice.

The results highlight challenges that are both general to PIC and specific to the care of persons living with SDS as they describe how trauma experiences typical for SDS (e.g., powerlessness, being met with disbelief, unsafety and unpredictability) can be reenacted in PIC, creating an increased vulnerability. Not all harmful or stressful experiences constitute trauma (Kleber, 2019). Trauma has been described as an external event that results in enduring effects on a person's well-being and involves both actual and perceived threats (Sweeney et al., 2018). It has further been argued that trauma experiences undermine a person's ability to manage their life and as events that exceed efforts to maintain control (Kleber, 2019). Our results detail how participants experienced multiple aspects of PIC, including the ward environment, unpredictable practices and encounters with staff who seemingly lack a trauma-informed approach, as distressing and harmful, and explicitly and implicitly resembling previous trauma experiences and provoking strong negative reactions. Further, replication of prior trauma dynamics may occur within care settings. Persons with traumatic experiences may be more likely to experience treatment practices as harmful or retraumatizing when care evokes feelings of powerlessness, entrapment or lack of involvement in decisions regarding their own care. Such situations may contribute to experiences of trauma recurring, and care may consequently be perceived as unsafe or dangerous in ways that resemble prior traumatic experiences (Substance Abuse and Mental Health Services Administration, 2014). Given that persons with SDS are likely to have prior traumatic experiences, it is important to consider the possibility of harmful experiences also being retraumatizing. Potential retraumatizing experiences were reflected throughout our findings, where participants described feeling at the mercy of others, distrusted and rejected and lacking control. Thus, we suggest that these experiences should be understood as retraumatizing. These findings add to previous research describing how everyday elements of PIC, e.g., coercion, locked doors, ward rules and the environment itself, can be potential reminders of unsafety and disempowerment, thereby contributing to retraumatization (Hennessy et al., 2023). Hallett et al. (2025), who describe adverse PIC experiences as contributing to traumatic outcomes that are often not addressed, propose conceptualizing these experiences through the perspectives of the ecosystem (i.e., the physical environment, resources and people in the environment), systems (i.e., processes and transitions) and finally the person (i.e., violation of autonomy and traumatization). This conceptualization resonates with our findings, which indicate that retraumatization in PIC may occur through a range of environmental and relational dynamics and not solely through coercion, which is perhaps the most commonly recognized form of retraumatization. Thus, our findings align with previous research indicating that persons with trauma experiences are at risk of retraumatization in PIC (Hallett et al., 2025; Hennessy et al., 2023; Sweeney et al., 2018), as well as recent studies describing experiences of powerlessness, dehumanization and punishment in PIC (Hallett et al., 2025; Scholes et al., 2022; Tully et al., 2023). This is in line with previous research describing how patients' trauma backgrounds can be overlooked in care delivery, reinforcing the need for a trauma-informed approach (Hennessy et al., 2023).

Our findings that participants perceived staff to be questioning and uninformed might not be surprising since previous research has indicated that professionals in PIC often feel unprepared when interacting with persons living with SDS, which can compromise care delivery (Kumar et al., 2022; Snyder & Keepers, 2023). At the same time, there are also general challenges in PIC, and quality of care can depend on ward culture and how nurses experience their work (Hubbard et al., 2017; Wyder et al., 2017). Previous research has shown that nurses in psychiatric care are vulnerable to compassion fatigue and secondary traumatization due to exposure to emotional stress which puts them at risk of emotional numbing and distancing (Hubbard et al., 2017; Rivera-Kloeppel & Mendenhall, 2023). The complexity of nursing in PIC requires nurses to balance conflicting demands, which often results in a gap between nurses' ideals and their actual capacity to provide care (Gabrielsson et al., 2016; Molin et al., 2016; Söderberg et al., 2022). The dominance of the biomedical perspective, with its narrow focus on clinical recovery, may further constrain nurses' endeavours and ability to practice in a recovery-oriented manner, contributing to moral stress (Wyder et al., 2017). This calls for solutions capable of addressing both specific and systemic challenges. Hallett et al. (2025) stress the need to redesign PIC towards a model that prioritizes safety, dignity and respect, shifting from behaviour correction to holistic, recovery-oriented care and emphasizing nurses' responsibility to facilitate safety and empathic encounters in order to provide high-quality PIC. Reflective and supportive structures are needed to enable nurses to deliver high-quality PIC (Hubbard et al., 2017; Wyder et al., 2017) and to work in a person-centred, recovery-oriented way (Wyder et al., 2017). Arguably, TIC is integral to recovery-oriented PIC and offers a framework for responding to the behaviour of persons in PIC through collaborative, attuned relationships (Wilson et al., 2017). TIC promotes a broad understanding of trauma and facilitates reflections on how to deliver care in PIC that is sensitive to the dynamics of trauma (Isobel, 2016). Hallett et al. (2025) stress the importance of recognizing and addressing all aspects of potentially trauma experiences in PIC—not solely focusing on coercion—to significantly enhance PIC.

Living with SDS involves managing parts with different opinions, and the results include conflicting opinions of the familiarity of trauma and the complex and contradictory nature of safety. Nevertheless, we argue that previous research supports interpreting our findings through the lens of retraumatization.

### Study limitations and methodological rigour

Recruitment through the NGO's social media limited the sample to those engaged with the organization online. This poses a risk of recruiting participants with specific agendas or experiences, but also enabled the inclusion of participants who have processed their experiences and can reflect on them meaningfully. To ensure trustworthiness despite the substantial volume and complexity of the data, the data were analyzed systematically through iterative steps. Results are presented with quotes from the participants to illustrate and strengthen the credibility of the analysis (cf. Graneheim et al., 2017; Lindgren et al., 2020). From a trauma-informed research perspective that emphasize staying grounded in the data and centreing the participants' voices (Isobel, 2021), analytical rigour was strengthened through a combination of continuous reflection among all authors, along with active and ongoing participation from the NGO representative. Several possible interpretations were explored and critically evaluated before choosing the final interpretation, which was considered to provide the most accurate and meaningful representation of the participants' experiences. Although transcripts were not returned to participants, we believe user involvement contributed to validating the interpretations and enhancing the credibility and confirmability of the analysis. While structuring results on the basis of positive versus negative experiences of care might have been perceived as providing a more balanced account, we chose to integrate positive and negative experiences within themes. We believe this better reflect the overall meaning and complexity of the data as seemingly positive experiences often contained contradictions and complicating factors. Thus, our results capture the contradictory nature of participants' experiences, rather than separating them based on valence. Similarly, Graneheim et al. (2017) describe how a theme consists of an essence of meaning which runs through the data, sometimes in the foreground and sometimes in the background. Additionally, Lindgren et al. (2020) highlight the importance of understanding both the data and the informant to make sense of both the words spoken and the person who said them.

## Conclusion

The findings indicate that persons living with SDS experience PIC as being stuck between a rock and an unnecessarily hard place, leaving them with the choice of dealing with life on their own or risking further harm in PIC. These findings contribute to a growing base of knowledge of how persons living with SDS are at risk of being harmed instead of helped when receiving care in PIC, as well as highlighting PIC's potential as a source of support, safety and healing. Previous research supports interpreting these results through the lens of retraumatization, suggesting a gap in awareness and understanding of trauma dynamics in everyday care. This supports the notion that there is a need for increased trauma awareness in mental health nursing in PIC as well as a need for deeper understanding of how this can best be achieved.

### Implications for practice

Given the focus of the present study and its grounding in mental health nursing, the implications discussed in this section primarily concern everyday nursing practice in PIC, rather than specific treatment interventions. Mental health nurses are positioned to lead the promotion of safety, dignity and respect in a trauma-informed way for persons living with SDS and they play a critical role in identifying persons' trauma experiences and integrating them into care planning. In PIC, nurses' capacity to recognize trauma-related needs is essential for delivering safe, person-centred and trauma-informed care and it requires recognition. For this, mental health nurses need authority, education, support, supervision and resources. This research is relevant for mental health nursing practice as it addresses the gap between the intended principles of person-centred and recovery-oriented care in PIC and the diverse realities experienced by patients. Persons living with SDS are vulnerable to being misunderstood and receiving suboptimal care and these discrepancies can affect recovery and lead to harmful outcomes. By generating knowledge about their experiences, this research provides insights to guide the development of a more responsive, safe and recovery-oriented mental health nursing practice.

## Supplementary Material

Supplementary Material COREQ_Checklist_3.pdf

## Data Availability

The participants of this study did not give written consent for their data to be shared publicly, so due to the sensitive nature of the research supporting data is not available. The data supporting the findings of this study are not publicly available due to privacy and ethical restrictions related to the protection of participant confidentiality. Access to de-identified data may be considered upon reasonable request. Requests should be directed to Anja Söderberg at anja.soderberg@ltu.se.
